# A Novel Missense Variant of *HOXD13* Caused Atypical Synpolydactyly by Impairing the Downstream Gene Expression and Literature Review for Genotype–Phenotype Correlations

**DOI:** 10.3389/fgene.2021.731278

**Published:** 2021-10-27

**Authors:** Ruiji Guo, Xia Fang, Hailei Mao, Bin Sun, Jiateng Zhou, Yu An, Bin Wang

**Affiliations:** ^1^Department of Plastic and Reconstructive Surgery, Shanghai Ninth People’s Hospital, Shanghai Jiao Tong University School of Medicine, Shanghai, China; ^2^Department of Anesthesiology and Critical Care Medicine, Zhongshan Hospital, Fudan University, Shanghai, China; ^3^Human Phenome Institute, MOE Key Laboratory of Contemporary Anthropology, and School of Life Sciences, Fudan University, Shanghai, China

**Keywords:** synpolydactyly, *HoxD13*, polyalanine extension, transcription regulation, genotype and phenotype

## Abstract

Synpolydactyly (SPD) is a hereditary congenital limb malformation with distinct syndactyly designated as SPD1, SPD2, and SPD3. SPD1 is caused by mutations of *HOXD13*, which is a homeobox transcription factor crucial for limb development. More than 143 SPD patients have been reported to carry *HOXD13* mutations, but there is a lack of genotype–phenotype correlation. We report a novel missense mutation of c. 925A > T (p.I309F) in an individual with atypical synpolydactyly inherited from her father with mild clinodactyly and three other different alanine insertion mutations in *HOXD13* identified by whole exome sequencing (WES) in 12 Chinese SPD families. Unlike polyalanine extension, which tends to form α-helix and causes protein aggregation in the cytoplasm as shown by molecular simulation and immunofluorescence, the c. 925A > T mutation impairs downstream transcription of *EPHA7*. We compiled literature findings and analyzed genotype–phenotype features in 173 SPD individuals of 53 families, including 12 newly identified families. Among the *HOXD13*-related individuals, mutations were distributed in three regions: polyalanine, homeobox, and non-homeobox. Polyalanine extension was the most common variant (45%), followed by missense mutations (32%) mostly in the homeobox compared with the loss-of-function (LOF) variants more likely in non-homeobox. Furthermore, a more severe degree and classic SPD were associated with polyalanine mutations although missense variants were associated with brachydactyly and syndactyly in hands and feet and LOF variants with clinodactyly in hands. Our study broadens the *HOXD13* mutation spectrum and reveals the profile of three different variants and their severity of SPD, the genotype–phenotype correlation related to the *HOXD13* mutation site provides clinical insight, including for genetic counseling.

## Introduction

Synpolydactyly (SPD) is a rare congenital limb deformity characterized by a fusion of adjacent digits and partial or complete digital duplication within the webs. In most patients, synpolydactyly affects the 3/4 fingers and the 4/5 toes (Malik and Grzeschik, 2010). Other features include brachydactyly or camptodactyly of the fifth finger and the fourth–fifth toes, clinodactyly, contraction and flexion of the interphalangeal joint, middle phalanx dysplasia of the second to fifth toes, and syndactyly of the 2/3 finger’s etc., (Brison et al., 2012b).

SPD is usually autosomal dominant with variable expressivity (Goodman et al., 1997). Most non-syndromic synpolydactyly cosegregates with a mutation in the *HOXD13* gene on chromosome 2q31 and is categorized as Synpolydactyly type 1 (SPD1, OMIM 186000), which is also known as syndactyly type II (SD II), according to the nomenclature of Temtamy and McKuisck (Temtamy and McKusick, 1978). Other rare genetic synpolydactyly comprises SPD2 (OMIM608180) caused by *FBLN1* gene mutation on chromosome 22q13.31 and SPD3 (OMIM610234), which is mapped to locus 14q11.2-q12 (Malik et al., 2006; Malik and Grzeschik, 2010).

*HOXD13* is a member of the *HOX* gene family (Laufer et al., 1994; Deng et al., 2015) expressed in the trunk, limb buds, and genital tubercles. The *HOX* gene cluster encodes a highly conserved transcription factor that plays an important role in morphogenesis during embryonic development (Von Allmen et al., 1996; Goodman, 2002). In humans, there are 39 *HOX* genes arranged in four different gene clusters (*HOXA*-*HOXD*) on chromosomes 7p14, 17q21, 12q13, and 2q31. The *HOXD13* gene is located at the 5’ end of the *HOXD* gene cluster and expressed later than the ones at the 3′ end of the clusters. It is involved in the regulation of distal and posterior regions of the emerging limb bud (Dolle et al., 1991; Brison et al., 2012a, b, 2014; Zhou et al., 2013). The *HOXD13* gene consists of two exons and encodes a protein of 343 amino acids (NM_000523.3). The functional domain of *HOXD13* consists of a 45 bp imperfect trinucleotide repeat, which encodes a 15-residue polyalanine tract within exon 1, a highly conserved 180 bp homeobox in exon 2, and a DNA-binding motif in the protein’s C-terminal region. The reported mutations in the *HOXD13* gene comprise three groups: polyalanine tract expansion, truncated mutations, and missense variants. Those mutations have distinct molecular pathogenic mechanisms: (1) polyalanine tract expansion or contraction mutations lead to cytoplasmic retention and aggregation of mutant HOXD13 protein. *In vitro* studies reveal that the mutant protein acts in a dominant-negative manner as it leads to cytoplasmatic aggregates that consist of not only wild-type HOXD13 proteins, but also other polyalanine-containing proteins, such as HOXA13 or RUNX2 (Albrecht et al., 2004). (2) Loss-of-function (LOF) mutations, including frameshift and nonsense mutations, affect the functional domains, including the DNA-binding homeodomain. (3) Missense mutations within the homeobox region affect the transcriptional activation ability of HOXD13 (Dai et al., 2014). Heterozygote and homozygote *HOXD13* mutations are reported, and homozygote carriers manifest severe phenotype with a complex type of SPD (Muragaki et al., 1996; Ibrahim et al., 2016).

Considering the heterogeneous malformations, molecular diagnosis, and treatment need of SPD, we investigate the clinical manifestation of *HOXD13* mutations and their underlying pathogenic mechanisms. We analyzed phenotypes of 53 hereditary SPD families with *HOXD13* mutations, including 12 Chinese non-syndromic SPD families, to explore genotype–phenotype correlation. Furthermore, we investigated the pathogenic mechanisms of aggregate formation within the cells, polymerization-free energy changes, and transcription regulation caused by the *HOXD13* mutations.

## Materials and Methods

### Patient Recruitment and Sample Collection

Patients with non-syndromic SPD and their relatives were recruited. The hands and feet of all probands were radiographed. Phenotypes of family members were acquired by either orthopedist diagnoses or descriptions from the proband’s parents (Ni et al., 2018). After informed consent was obtained, peripheral blood samples of patients and their parents were collected for genomic DNA extraction using the DNeasy Blood and Tissue kit (Qiagen, CA 69506, Germany). This study was approved by the ethics committee of the Ninth People’s Hospital Affiliated to Shanghai Jiao Tong University (No. SH9H-2018-T50-2).

### Trio Whole Exome Sequencing

The genomic DNA samples were processed following the protocol of the SureSelectXT Target Enrichment System for the Illumina Paired-End Sequencing Library (Agilent, CA 95051, United States). Prepared samples were performed by next-generation sequencing (NGS) on a HiSeq system (Illumina). The average coverage was above 20×.

### Next-Generation Sequencing Data Analysis

The sequencing data were transformed into Fastq format, and the quality was evaluated by FastQC program. We used the BWA software (0.7.12) for sequence alignment with hg19 genomic reference. The single nucleotide variants and insertions and deletions (InDels) were detected by HaplotypeCaller. The variants were annotated by ANNOVAR software after filtering the variants with AF ≥ 1% and intronic and synonymous variants without splicing effects (Ni et al., 2018).

### Verification by Sanger Sequencing

The variants of the *HOXD13* gene identified in probands were validated and confirmed in the affected individuals by Sanger sequencing. The PCR system was performed using a PCR kit (Toyobo, CA KFX-101, Japan) in the reaction solution and condition referring to the manufacturer’s instructions. The primers were as follows: 1-1F GCGCAGCCAATGGCAC; 1-1R CTTCTCCACGGGAAAGCCTC; 1-2F CCTCTTCTGCCGTTG TAGCG; 1-2R TTAACCCTGGTCACGTGTGG; 2-1F ACT GTCCTCATGAACGTGCC; 2-1R GGCCTGGAGGGAGAAA CAAA.

### Genotype–Phenotype Relationship Analysis

A systematic review of literature and variant databases (ClinVar, Mastermind, HGMD, LOVD, DECIHPER) was conducted to curate the *HOXD13* variations. We selected relevant studies based on the following criteria: (1) having a *HOXD13* mutation site without other gene mutations, (2) having limb malformation phenotypes, (3) having a complete description of the clinical features of malformation. We used “SPD,” “polysyndactyly,” “synpolydactyly,” or “*HOXD13*” as keywords in searching PubMed. A total of 191 studies with *HOXD13* mutations and 84 studies with phenotype and *HOXD13* mutations were retrieved. After excluding 75 duplicates in the two groups and removing those missing photographs of relevant phenotypes, the previously reported mutations and the findings in our study of 12 families were used for statistical analysis (Supplementary Material 1).

To evaluate the severity of SPD, we defined an SPD severity score, which is a summary of the frequency of syndactyly, polydactyly, clinodactyly, camptodactyly, and brachydactyly in one patient. One point was assigned to per trait except for synpolydactyly, which was given a score of two points due to its combination of syndactyly and polydactyly. A higher SPD severity score means more limb defects. The statistical analysis was performed on the relationship between the SPD severity score and the *HOXD13* variant type and location.

### Plasmid Construction and Transfection

*HOXD13* wild type and c.925A > T (p.I309F) DNA fragments were amplified by PCR and cloned into pcDNA3.1(+) using the LIC ligase system (NEB system). *HOXD13* poly-Ala mutant plasmid with + 7A, + 8A, and + 9A (p.A65_A71dup, p.A64_A71dup, p.A63_A71dup) were constructed by inserting synthesized oligo nucleotides encoding the additional alanines (Generay Biotech Co., Ltd., Shanghai) and confirmed by Sanger sequencing. A 660bp fragment of *EPHA7* gene promoter containing a HOXD13 binding site (from −580 to + 80) was obtained from human genomic DNA by PCR and inserted into the *Xho*I and *Hin*dIII sites of a pGL3-basic vector (Promega, E1751, United States) to generate a pGL3-EPHA7 reporter construct (Zhao et al., 2007; Dai et al., 2014). COS-7 cells were cultured in DMEM/high-glucose medium (Hyclone, CA SH30022.01, United States) supplemented with 1% penicillin/streptomycin and 10% fetal bovine serum (6 × 10^5^ cells per well in a six-well plate, 1 × 10^5^ cells per well in a 24-well plate). After the cells were incubated at 37°C for 24 h, 50 μL of DNA-lipid complex (DNA: Lipofectamine^®^ 2000 Reagent, 250ng: 1μL) (Invitrogen, Lipofectamine^®^ 2000 Reagent, United States) was added per well in a 24-well plate for immunofluorescence detection, and 250 μL DNA-lipid complex was added per well in a six-well plate for luciferase assay.

### Immunofluorescence Assay

COS-7 cells transfected with pcDNA3.1-HOXD13^WT^, pcDNA3.1-HOXD13^I309F^, pcDNA3.1-HOXD13^+7A^, pcDNA3.1- HOXD13^+8A^, or pcDNA3.1- HOXD13^+9*A*^ were incubated for 1 day at 37°C. Cells were treated as follows: washed with PBS and fixed with 4% paraformaldehyde for 10 min, permeabilizied with 0.2% Triton X for 10 min, and blocked with 1% bovine serum albumin for 0.5 h; the cells were incubated with anti-HOXD13 primary antibody for 1 h at 37°C (Abcam, CA ab19866, United States) (1:100 dilution) and FITC conjugated secondary antibody for half an hour at 37°C (Abcam, CA ab6717, United States) (1:200 dilution). The nuclei were stained by DAPI and observed under the fluorescent microscope in the darkroom after mounting.

### Luciferase Assay

PcDNA3.1-HOXD13^WT^ and pcDNA3.1-HOXD13^I309F^ were cotransfected with Renilla and pGL3-EPHA7 luciferase reporter plasmid into 293T cells and incubated for 24 h at 37°C. The cells were lysed following the protocol of the Dual-Luciferase^®^ Reporter Assay (Promega, CA E1960, United States): 100 μl of LAR II was predispensed into the luminometer tube before programming the GloMax 20/20 single tube luminometer, 20 μl of PLB lysate was transferred into a tube, and firefly luciferase activity was measured. Then, 100 μl of Stop and Glo^®^ Reagent was dispensed, and renilla luciferase activity was measured. The assay was repeated three times.

### Protein Structure Prediction and Molecular Simulation

I-TASSER online software^1^ was used to simulate the 3-D structure of HOXD13 homeodomain. The amino acid residues between 276 and 335 were entered to cover the location of the I309F mutation. In addition, we performed molecular dynamics (MD) simulation of the + 0A, + 7A, + 8A, and + 9A monomer using the Gromacs (V5.1.2) software with Charmm36 force field parameter and TIP3P model to figure out the binding energy between two dimers.^2^ The initial structure model of dimer and steered molecular dynamics (SMD) simulation were carried out based on the stable conformation of MD simulation equilibrium. Umbrella sampling following SMD simulation and the weighted histogram analysis method were employed to obtain the potential mean force (PMF) during the depolymerization of the polyalanine dimer, which was used to indicate the polymerization-free energy.

### Statistical Analyses

Statistical analyses were performed with the GraphPad Prism 8 software. The differences in the relative luciferase activity were assessed by unpaired *t*-test, which was also applied to analyze the differences in severity scores between polyA and homeobox, offspring and parents, among different expansion sizes of polyalanine tract. The statistical differences in severity scores between homeobox and non-homeobox and between polyA and non-homeobox were assessed by Mann–Whitney test due to the unequal variances. *P* < 0.05 was considered statistically significant.

## Results

### Clinical Features and *HOXD13* Mutations

**FIGURE 1:**
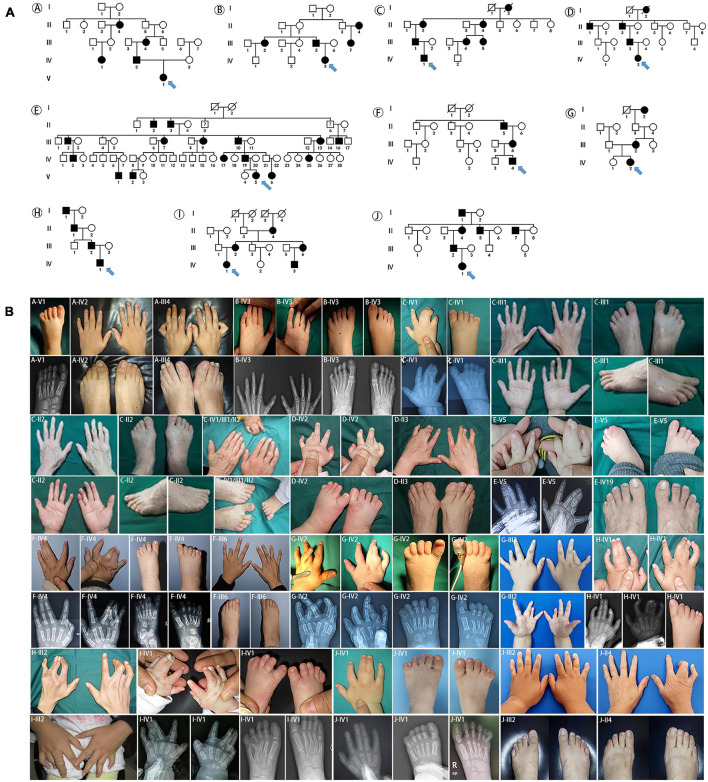
Pedigrees and clinical phenotypes in SPD families with *HOXD13* polyalanine extension. **(A)** The relationship charts of 10 families with synpolydactyly. **(B)** Clinical manifestations of the limbs in affected individuals, including synpolydactyly, syndactyly, syndactyly, clinodactyly, camptodactyly, brachydactyly.

**FIGURE 2:**
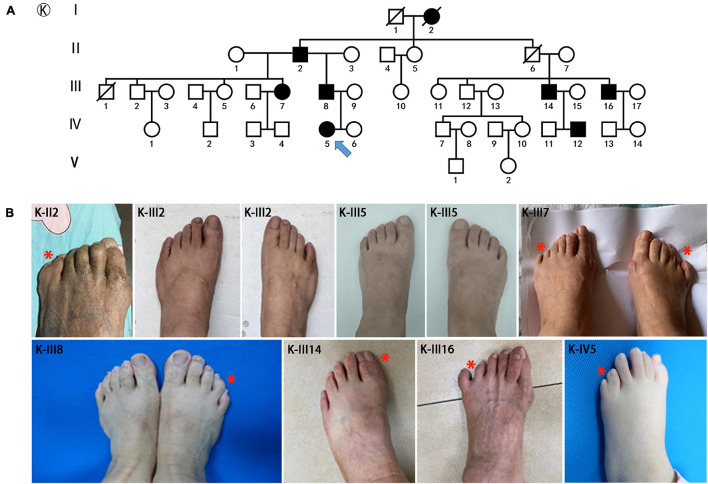
Pedigrees and clinical phenotypes in SPD families with *HOXD13* c.925A > T mutation. **(A)** The relationship chart of the family with atypical synpolydactyly. **(B)** Clinical manifestations of the limbs in affected individuals, including postaxial and preaxial polydactyly, camptodactyly.

A total of 12 families with *HOXD13* variants were collected from our clinical center. The pedigree investigation and imaging or X-ray of hands or feet are described in Figures 1, 2, including one pedigree (No 32 in Supplementary Table 1), which was reported previously (Ni et al., 2018). Besides synpolydactyly as the typical manifestation, the patients were often accompanied with other phenotypes, including syndactyly (3/4 fingers, 1/2 or 2/3 or 4/5 toes), polydactyly (1/2 fingers, 1/2 or 5/6 toes), clinodactyly (2nd or 4th or 5th finger, 1st or 2nd or 4th or 5th toe), camptodactyly (all five fingers, 1st or 3rd or 4th toe), and brachydactyly (all five fingers, 1st or 2nd or 3rd or 4th toe). Syndactyly presented in an SPD patient was cutaneous or bony. The well-developed duplicated toe could be found at the tibial or fibular side. Clinodactyly can be represented as delta or trapezoidal phalanx.

The major mutations of *HOXD13* observed in this study were polyalanine expansions in exon 1. We identified c.192_212 alanine insertion mutation of *HOXD13* in five families (Family A/B/C/D/E), c.189_212 alanine insertion mutation in Family F and c.186_212 alanine insertion mutation in four families (Family H/I/J/K) (Supplementary Material 1). We also found a frameshift mutation of c.610delC inherited from the affected father, which led to a truncated protein (p.P204fs*61) reported previously (Ni et al., 2018). Another novel missense mutation of c.925A > T (p.I309F) was identified in the proband of Family K (Figure 2), which was inherited from her father with mild camptodactyly of the fifth digit of right foot.

### Genotype–Phenotype Relationship Analysis

To expand our genotype–phenotype analysis, we compiled literature findings and included 53 families with *HOXD13* mutations in this study. Of these *HOXD13* mutations, polyalanine tract expansion was observed in 11 reports, missense mutations in 12 reports, nonsense mutations in 13 reports, and deletion and insertion mutations in five reports. Among the 53 families, polyalanine extension was the most common form of mutation (24/53, 45%) in *HOXD13*. The second most common form of variants was missense mutation (17/53, 32%). The loss of function variants was identified in 10 families (10/53, 19%). Of 10 variants located in the homeobox domain of *HOXD13*, eight of them (8/10, 80%) were missense mutations, and of 12 variants located in the non-homeobox domain of HOXD13, eight of them (8/12, 67%) were truncating mutations.

**FIGURE 3:**
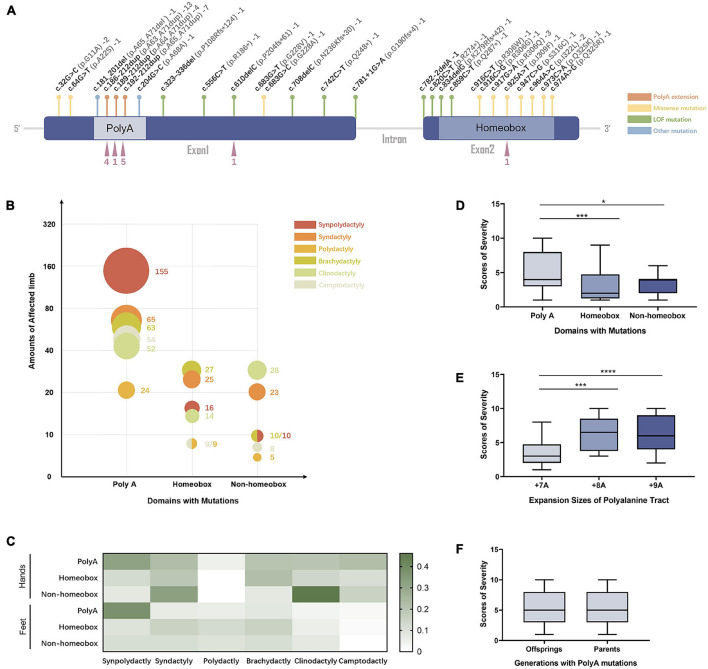
The genotype–phenotype relationship in SPD patients with *HOXD13* mutation. **(A)** The mutation sites in *HOXD13*. **(B)** The number of affected limbs with different phenotypes in different domains with *HOXD13* mutation. **(C)** Heat map of the phenotype:genotype ratios in different *HOXD13* mutation regions in hands and feet separately. **(D)** The scores of severity in different domains with *HOXD13* mutation. **(E)** The scores of severity with different *HOXD13* expansion sizes of polyalanine tract. **(F)** The scores of severity in the offsprings and parents who both have polyA mutations in the *HOXD13* gene.

The 27 curated variants from our expanded data set were mapped to different regions of the *HOXD13* gene (Figure 3A). In patients with *HOXD13* polyA mutations, synpolydactyly was observed in 155 affected hands and feet (Figure 3B) and especially in feet (> 35%) (Figure 3C). We further compared the clinical features of patients with variants in three functional regions of *HOXD13* (polyA, homeobox, and non-homeobox). We observed that the patients with *HOXD13* mutations in the homeobox region had the tendency to present brachydactyly, especially in hands. The patients with non-homeobox mutations in *HOXD13* mostly presented clinodactyly in hands (Figures 3B,C). Due to anatomical differences between hand and foot, clinodactyly was often observed in fingers with a greater range of motion and less often in toes.

Statistical analysis was performed on the correlation between the SPD severity scores and *HOXD13* variation types. A statistically significant difference in severity scores between polyA mutation and the variants in homeobox (*P* < 0.001) or non-homeobox (*P* < 0.05) was observed, and no significant difference was found between homeobox and non-homeobox variations (Figure 3D). The severity scores between polyalanine tract with + 7A expansion and + 8A or + 9A were statistically different (*P* < 0.001) although the difference between + 8A and + 9A was insignificant (Figure 3E). When we examined the transmission effect of poly-Ala expansion, there was no statistically significant difference in severity scores between the offspring and their parents (*p* > 0.05) (Figure 3F), suggesting that their clinical manifestation severity remains the same.

### Cytoplasmic Aggregation Effect of HOXD13 Variants

**FIGURE 4:**
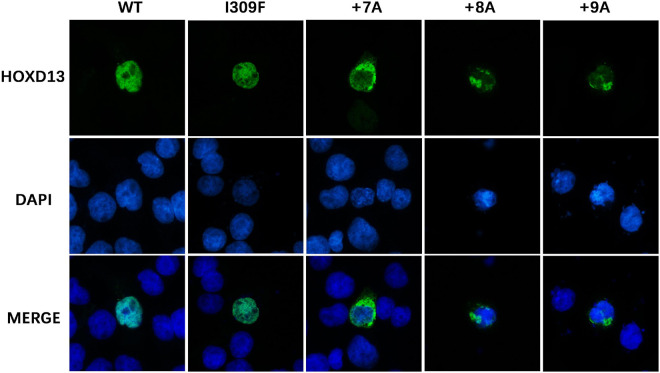
Subcellular location of HOXD13 protein. The blue color in the nucleus shows DAPI staining, and the green color shows HOXD13 staining. WT and I309F mutated HOXD13 protein are localized in nucleus only. Mutants with + 7A, + 8A, + 9A inserted in the polyalanine fragment partially entered the nucleus.

To investigate the effect of *HOXD13* mutation on protein aggregation in the cytoplasm, we transfected COS-7 cells with plasmids carrying the wild type, polyalanine extension (+ 7A, + 8A, + 9A), or I309F variation of the *HOXD13* gene. We found the polyalanine expansion mutant (+ 7A, + 8A, + 9A) was partially localized in the cytoplasm, and the wild type and I309F mutant were localized in the nucleus. This indicates that I309F mutant protein did not interfere with nuclear transport of the protein, but the polyalanine extension mutant proteins resulted in cytoplasmic aggregates (Figure 4).

### Polyalanine Extension Led to Structural Change

**FIGURE 5:**
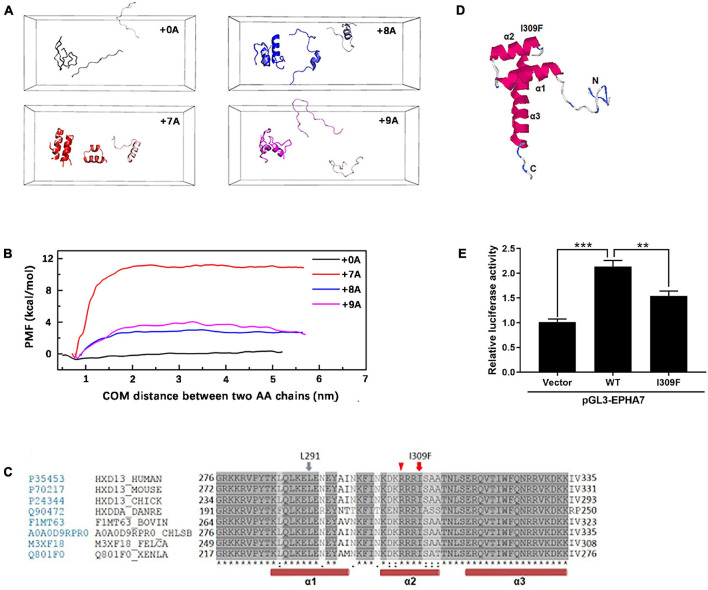
Molecular simulation of mutant HOXD13 and the transcriptional activity of I309F. **(A)** The simulated conformation of the polyalanine fragment dimer. Gray: 15 alanine (+ 0A), Red: 22 alanine (+ 7A). Blue: 23 alanine (+ 8A). Purple: 24 alanine (+ 9A). **(B)** The polymerization energy of alanine dimer. The PMF of 15, 22, 23, 24 alanine was 1.15, 11.71, 3.15, 4.58 kcal/mol, respectively. **(C)** I309 is located in the second α-helix of the homeobox domain. **(D)** The computed 3-D structure of the HOXD13 homeobox domain was generated by the I-TASSER online software. **(E)** Transactivation activity of the pGL3-EPHA renilla luciferase reporter plasmid by HOXD13 I309F mutants and WT. The results of the luciferase assay are presented as relative luciferase activity.

Molecular simulation shows that the dimer with alanine insertion mutations (+ 7A, + 8A, + 9A) had conformation changes at the distances of 1.0, 3.0, and 5.5 nm, respectively, and presented varying degrees of α-helix (Figure 5A) compared with the one without alanine insertion (+ 0A), which remained free as β-sheet or random coil structure. The polymerization energy of polyalanine dimer with alanine insertion mutation was higher than the wild type, suggesting that proteins with alanine insertion could not depolymerize easily and tended to aggregate (Figure 5B).

### I309F Reduced the Transcriptional Activation of HOXD13

Based on the computed 3-D structure of HOXD13, I309F is located in the second α-helix of the homeobox domain, which is directly involved in DNA binding (Figure 5C). Because the parallel localization of the first and second α-helices is important for DNA binding to the third helix and N-terminal tail (Figure 5D), the replacement of isoleucine with a larger phenylalanine residue may disrupt its interaction with the L291 residue of the second α-helix, which results in dysfunction of HOXD13.

We performed luciferase assays of the luciferase reporter gene driven by the promoter of *EPHA7*, which is a downstream gene of HOXD13. The transcriptional activation ability of HOXD13 was examined to understand the regulation effect of the HOXD13 I309F variation. In comparison with the wild-type HOXD13, we observed that the luciferin signal of EPHA7 was significantly decreased in the I309F transfectants. This indicates an impaired transcription factor activity of I309F mutant on the downstream target gene *EPHA7* (Figure 5E).

## Discussion

SPD is a rare limb deformity showing a special combination of polydactyly and syndactyly (Malik and Grzeschik, 2010). *HOXD13* is an important genetic factor of SPD. There is variable and asymmetrical expressivity of clinical phenotypes caused by *HOXD13* mutations (Malik and Grzeschik, 2010; Al-Qattan, 2020) and studies on genotype–phenotype correlation are limited.

The alanine insertion mutations at c.192_212, c.189_212, and c.186_212 identified in this study are located in the protein-coding region of the first exon of *HOXD13*. So far, a total of nine kinds of human diseases were reported to be associated with alanine repeat mutations, including SPD (*HOXD13*) (Goff and Tabin, 1997), blepharophimosis syndrome (*FOXL2*) (Baere et al., 2003), cleidocranial dysplasia (*RUNX2*) (Mundlos et al., 1997), congenital central hypoventilation syndrome (*PHOX2b*) (Jeanne et al., 2003), holoprosencephaly type 5 (*ZIC2*) (Brown et al., 2001), hand-foot-genital syndrome (*HOXA13*) (Boris et al., 2002), growth hormone deficiency (*SOX3*) (Laumonnier et al., 2002), Paddington syndrome (*ARX*) (Petter et al., 2002), and oculopharyngeal muscular dystrophy (*OPMD*, *PABPN1*) (Brais et al., 1998). All polyalanine repeats are believed to disrupt protein folding, leading to intracellular aggregation (Albrecht et al., 2004). The alanine expansion in HOXA13, RUNX2, and SOX3 resulted in the accumulation of protein in the cytoplasm with interaction with heat shock proteins (Albrecht et al., 2004; Villavicencio-Lorini et al., 2010). Therefore, the protein aggregation due to the alanine extension mutation could prevent the translocation of transcription factor HOXD13 protein into the nucleus resulting in the dysregulation of downstream genes. The length of Ala expansions was previously reported to correlate with the HOXD13 aggregation, and the mutant protein with + 14 Ala and + 21 Ala were exclusively found in the cytoplasm (Albrecht et al., 2004). The alanine repeat expansions resulted in reduced HOXD13-dependent transcriptional activity without affecting DNA binding (Basu et al., 2020). We observed that the alanine extension mutant HOXD13 proteins (+ 7A, + 8A, + 9A) were partially localized in the cytoplasm (Figure 4). Furthermore, the molecular simulation of the alanine fragment dimer showed that the molecular structure and polymerization energy were changed when the extra alanine residues were inserted to the 15 alanine polypeptides, and the dimer was more likely to form α-helical structures and aggregation (Figures 5A–D). This indicates that the repeat expansion changed the biophysical properties of HOXD13 protein.

We identified a novel missense mutation c. 925A > T (p.I309F) in the homeodomain of *HOXD13*. The mutant protein was localized in the nucleus with no aggregation in the cytoplasm. The homeobox domain contains 60 residues and is the key region for HOXD13 to bind to DNA as a transcription factor (Scott et al., 1989; Johnson et al., 2003; Burglin and Affolter, 2016). The amino acid sequence of the homeobox domain is highly conserved among different genes in many organisms (Scott et al., 1989; Kissinger et al., 1990). We investigated the effect of a I309F mutation on the transcription activation of the EPHA7 gene, which is one of the downstream genes of HOXD13 and plays an important role in limb development (Valentina and Vincenzo, 2006). Reduced transcription activation of EPHA7 was observed in cells transfected with c. 925A > T *HOXD13*, indicating that the I309F mutation in the homeobox region resulted in impaired transcriptional activation (Figure 5E). This is consistent with other studies that report the effect of other missense mutations in the homeobox domain, such as c.940A > C (p.I314L) and c.893G > A (p. R298Q) (Johnson et al., 2003; Wang et al., 2012). In this study, the girl who carried the c. 925A > T mutation had polydactyly L5/6 toes, and this was inherited from the affected father who had a mild phenotype with camptodactyly at his right foot. In this four-generation family, there were seven affected individuals who had variable expressivity of atypical SPD at B5/6 or L5/6 toe in feet with HOXD13 c. 925A > T mutation. Considering the segregation evidence of the family (Figure 2), the variant c. 925A > T (p.I309F) is pathogenic according to ACMG Guidelines [PM2_P, PS3_M (reduce the transcription of EPHA7), PP1_M (6 affected segregations), PM1 (in homeodomain), PP3 (REVEL score 0.95)].

It is shown that the mutations of *HOXD13* led to variable expressivity and a broad spectrum of clinical features (Brison et al., 2014). We summarized the phenotypes of affected individuals in 53 families with the *HOXD13* mutation spectrum to understand the potential correlation (Supplementary Table 1). Although the variable phenotypes were observed in the individuals with *HOXD13* heterozygous mutations, the defects were only related to limbs, a single organ system, and seldom syndrome although *HOXA13* mutations were associated with hand-foot-genital syndrome (Mortlock and Innis, 1997). Among all the clinical features, six of them were common limb abnormalities (synpolydactyly, syndactyly, polydactyly, clinodactyly, camptodactyly, and brachydactyly), and synpolydactyly in the 3/4 fingers and the 4/5 toes were the primary phenotypes. In addition, abnormal flexion crease, stiff joint, broad halluce, symphalangism, oligodactyly, and other features are also described. The cortical bone morphology was abnormal in the individuals with *HOXD13* mutations (Villavicencio-Lorini et al., 2010), resulting in the irregular shape of the long bones in the hand and foot.

The 27 variants from our compiled data set were mapped to the *HOXD13* gene (Figure 3A) and categorized into three types of mutations based on the molecular pathogenic mechanisms: polyananine extension, missense mutation, and LOF mutation. The most common mutations, i.e., the polyalanine extension, were frequently associated with classic SPD (Zhao et al., 2007). According to the severity evaluation in this study, patients with polyalanine tract expansion mutations had more severe manifestations than the ones with LOF or missense mutations. In patients with *HOXD13* polyalanine tract expansion mutations, synpolydactyly was observed in 155 affected hands and feet (Figure 3B) and especially in feet (> 35%) (Figure 3C). Meanwhile, they were also likely to present with syndactyly, brachydactyly, and camptodactyly in hands (> 20%). The misfolding and cytoplasmic aggregation of polyalanine expansion mutants were conferred as dominant negative phenotypes. The missense mutants tend to localize in the homeobox region although the LOF (frameshift or nonsense) mutants usually reside in the non-homeobox region. The patients with LOF mutations in the non-homeobox region had *a priori* clinodactyly (> 40%) in hands as joints have a wider range of motion than feet. The LOF mutations in the homeobox region could be dominant LOF of transcription activity of HOXD13 owing to the truncated proteins produced. The missense mutations in the homeobox region were often presented as brachydactyly and syndactyly in hands and feet. The missense mutations, such as the c. 925A > T (p.I309F) mutation, had a deleterious effect on the transcriptional activation of the human *EPHA7* promoter without forming aggregates in the cytoplasm. For missense variants outside the homeobox region of *HOXD13*, the expression of the HOXD13 G220V mutant was studied in chick limb by Fantini et al. and also presented LOF with the impaired capability to bind DNA and to activate the downstream target of HOXD13 (Fantini et al., 2009). Another G11A missense mutation at the position 32 of the coding region where HOXD13 binds with *GLI3R* resulted in the same phenotype as the depletion of *GLI3R* (Chen et al., 2004), suggesting a new molecular mechanism of *HOXD13*.

We thoroughly evaluated the correlation of SPD phenotype with genotype of *HOXD13* in this study. Previous reports show that homozygous patients may present severe phenotypes but not all (Ibrahim et al., 2016). We created a severity score for the evaluation of synpolydactyly. By comparing the clinical features in the SPD patients with different *HOXD13* mutations (PolyA, homeobox, and non-homeobox), we found a statistically significant difference in the severity scores between polyA mutation and homeobox or non-homeobox (*P* < 0.05) although no significant difference was observed between the variants in homeobox and non-homeobox (Figure 3D). In addition, a difference was statistically significant between the polyalanine tract with + 8A or + 9A expansion or + 7A (*P* < 0.001) (Figure 3E). It suggests that the individuals with polyA mutations, especially + 8A or + 9A expansion, might have more severe phenotypes, and the ones carrying the missense mutations might have atypical SPD. Penetrance of these variants could not be evaluated due to the lack of genotype in the unaffected.

Our study analyzed the clinical characteristics and inheritance of SPD caused by *HOXD13* mutations and confirmed that the HOXD13 abnormality plays an important role in SPD. We first revealed correlations between the location and types of mutations of HOXD13 and severities of the phenotypes by the overview of 173 affected individuals with *HOXD13* mutations. We compared the mechanism of different types of mutations in *HOXD13* and indicate that the alanine insertion mutations lead to protein aggregation in the cytoplasm, and the mutations in the homeobox decreased downstream transcriptional activity. The finding is of value for a better understanding of the variable phenotypes and clinical utility of HOXD13-related disease.

## Data Availability Statement

The datasets presented in this study can be found in online repositories. The names of the repository/repositories and accession number(s) can be found in the article/Supplementary Material.

## Ethics Statement

The studies involving human participants were reviewed and approved by the Independent Ethics Committee, the Ninth People’s Hospital Affiliated to Shanghai Jiao Tong University School of medicine. Written informed consent to participate in this study was provided by the participants’ legal guardian/next of kin.

## Author Contributions

BW, YA, and RG: conceptualization. RG, XF, YA, and BW: data curation. RG and YA: formal Analysis. BW, YA, and HM: funding acquisition. RG, XF, HM, BS, JZ, and YA: methodology. RG, XF, and HM: software. RG and HM: visualization. RG: writing – original draft. BW and YA: writing – review and editing. All authors contributed to the article and approved the submitted version.

## Conflict of Interest

The authors declare that the research was conducted in the absence of any commercial or financial relationships that could be construed as a potential conflict of interest. The reviewer TY declared a shared affiliation with several of the authors, RG, JZ, XF, BS, and BW, to the handling editor at the time of review.

## Publisher’s Note

All claims expressed in this article are solely those of the authors and do not necessarily represent those of their affiliated organizations, or those of the publisher, the editors and the reviewers. Any product that may be evaluated in this article, or claim that may be made by its manufacturer, is not guaranteed or endorsed by the publisher.

## Footnotes

1
https://zhanglab.ccmb.med.umich.edu/I-TASSER/

2
https://www.gromacs.org/

## Supplementary Material

Supplementary Material 1 *HOXD13* mutation sites in SPD patients. **(A)** Sanger sequencing results in patients with polyalanine extension (c.192_212dup, c.189_212dup, and c.186_212dup). **(B)** Sanger sequencing results in the family members with c.925A > T mutation.
