# Supplementary material for: A Novel Missense Variant of HOXD13 Caused Atypical Synpolydactyly by Impairing the Downstream Gene Expression and Literature Review for Genotype–Phenotype Correlations
Source: Front Genet. 2021 Oct 27;12:731278. doi: 10.3389/fgene.2021.731278 (PMC8579070; doi:10.3389/fgene.2021.731278)
Supplement: Supplementary Material 1 — HOXD13 mutation sites in SPD patients. (A) Sanger sequencing results in patients with polyalanine extension (c.192_212dup, c.189_212dup, and c.186_212dup). (B) Sanger sequencing results in the family members with c.925A > T mutation. [file Table_1.DOCX]

| **Table 1. The phenotype and genotype related to HOXD13 mutation.** | | | | | | | | |  |  |
| --- | --- | --- | --- | --- | --- | --- | --- | --- | --- | --- |
| **Family No.** | **Sample No.** | **Change of NM‡** | **Change of Aa** | **Functional Domain** | **Phenotype** | | | | **Ref.†** | **Reported Patient No.** |
|  |  |  |  |  | **Hands*** | **Feets*** | **Severity Score** | |  |  |
| 1 | S1 | c.32G>C | p.G11A | Non-homeobox | Syndactly R3/4 | Normal | | 1 | Xie, F. 2011 | Case10 |
| 2 | S2 | c.32G>C | p.G11A | Non-homeobox | Camptodactyly B5 | Brachydactyly B5 | | 4 | Brison, N. 2012 | IV1 |
| 3 | S3 | c.64G>T | p.A22S | Non-homeobox | Normal | Syndactly L4/5/6 | | 2 | Xie, F. 2011 | Case17 |
| 4 | S4 | c.181_201del (reported as c.157_177del) | p.A65_A71del (reported as p.A53_A59del) | PolyA | Brachydactyly B3/4/5 | Brachydactyly B3/4/5 | | 4 | Zhao, X. 2007 | Family2 IV19 |
|  | S5 | c.181_201del (reported as c.157_177del) | p.A65_A71del (reported as p.A53_A59del) | PolyA | Brachydactyly B4/5 | Normal | | 2 | Zhao, X. 2007 | Family2 IV23 |
|  | S6 | c.181_201del (reported as c.157_177del) | p.A65_A71del (reported as p.A53_A59del) | PolyA | Brachydactyly B1-5 | Brachydactyly B2/3 | | 4 | Zhao, X. 2007 | Family2 III10 |
|  | S7 | c.181_201del (reported as c.157_177del) | p.A65_A71del (reported as p.A53_A59del) | PolyA | Brachydactyly B3/4/5 | Brachydactyly B2/3 | | 4 | Zhao, X. 2007 | Family2 V5 |
| 5 | S8 | c.192_212dup | p.A65_A71dup | PolyA | Normal | Synpolydactyly L5/6 | | 2 | Our study (Family A) | V1 |
|  | S9 | c.192_212dup | p.A65_A71dup | PolyA | Clinodactyly B5 | Normal | | 2 | Our study (Family A) | IV2 |
|  | S10 | ND | ND | ND | Clinodactyly B5 | Syndactyly R4/5 | | 3 | Our study (Family A) | III4 |
| 6 | S11 | c.192_212dup | p.A65_A71dup | PolyA | Syndactly B3/4 | Synpolydactyly B5/6 | | 6 | Our study (Family B) | IV3 |
| 7 | S12 | c.192_212dup | p.A65_A71dup | PolyA | Synpolydactyly R3/4 | Synpolydactyly R5/6 | | 4 | Our study (Family C) | IV1 |
|  | S13 | c.192_212dup | p.A65_A71dup | PolyA | Synpolydactyly R3/4, Clinodactyly B5 | Synpolydactyly B5/6 | | 4 | Our study (Family C) | III1 |
|  | S14 | ND | ND | ND | Synpolydactyly R3/4, Clinodactyly B5 | Synpolydactyly B5/6 | | 8 | Our study (Family C) | II2 |
| 8 | S15 | c.192_212dup | p.A65_A71dup | PolyA | Synpolydactyly B3/4 | Synpolydactyly B5/6 | | 8 | Our study (Family D) | IV2 |
|  | S16 | c.192_212dup | p.A65_A71dup | PolyA | Synpolydactyly R3/4 | Normal | | 2 | Our study (Family D) | III3 |
|  | S17 | ND | ND | ND | Synpolydactyly B3/4 | Syndactyly R4/5 | | 5 | Our study (Family D) | II3 |
| 9 | S18 | c.192_212dup | p.A65_A71dup | PolyA | Synpolydactyly B3/4 | Synpolydactyly B5/6 | | 8 | Our study (Family E) | V5 |
|  | S19 | c.192_212dup | p.A65_A71dup | PolyA | Normal | Synpolydactyly B5/6 | | 4 | Our study (Family E) | IV19 |
| 10 | S20 | c.192_212dup | p.A65_A71dup | PolyA | Syndactyly R3/4 | Normal | | 1 | Malik, S. 2007 | VIII19 |
|  | S21 | c.192_212dup | p.A65_A71dup | PolyA | Syndactyly R3/4 | Normal | | 1 | Malik, S. 2007 | VIII20 |
|  | S22 | c.192_212dup | p.A65_A71dup | PolyA | Normal | Synpolydactyly B1/2 | | 4 | Malik, S. 2007 | VIII24 |
|  | S23 | c.192_212dup | p.A65_A71dup | PolyA | Polydactyly L3/4 | Normal | | 1 | Malik, S. 2007 | VIII35 |
|  | S24 | c.192_212dup | p.A65_A71dup | PolyA | Synpolydactyly B3/4/5 | Normal | | 8 | Malik, S. 2007 | VIII43 |
|  | S25 | c.192_212dup | p.A65_A71dup | PolyA | Polydactyly L3/4 | Normal | | 1 | Malik, S. 2007 | VIII44 |
|  | S26 | c.192_212dup | p.A65_A71dup | PolyA | Normal | Syndactyly L4/5 | | 1 | Malik, S. 2007 | VIII45 |
|  | S27 | c.192_212dup | p.A65_A71dup | PolyA | Normal | Synpolydactyly B5/6 | | 4 | Malik, S. 2007 | VIII50 |
| 11 | S28 | c.192_212dup | p.A65_A71dup | PolyA | Clinodactyly L5, Brachydactyly L5 | Synpolydactyly B5/6 | | 6 | Kjaer K.W, 2005 | Family1 VI15 |
|  | S29 | c.192_212dup | p.A65_A71dup | PolyA | Syndactyly R3/4 | Synpolydactyly R5/6 | | 5 | Kjaer K.W, 2005 | Family1 V13 |
|  | S30 | c.192_212dup | p.A65_A71dup | PolyA | Syndactyly R3/4 | Syndactyly R4/5 | | 2 | Kjaer K.W, 2005 | Family1 V16 |
|  | S31 | c.192_212dup | p.A65_A71dup | PolyA | Brachydactyly R1,B5 | Normal | | 2 | Kjaer K.W, 2005 | Family1 VI29 |
|  | S32 | c.192_212dup | p.A65_A71dup | PolyA | Brachydactyly R1 | Polydactyly B5/6 | | 3 | Kjaer K.W, 2005 | Family1 V21 |
|  | S33 | c.192_212dup | p.A65_A71dup | PolyA | Syndactyly B3/4, Clinodactyly R5, L3, Brachydactyly B2,5 | Polydactyly B5/6 | | 8 | Kjaer K.W, 2005 | Family1 VII3 |
|  | S34 | c.192_212dup | p.A65_A71dup | PolyA | Normal | Synpolydactyly B5/6 | | 4 | Kjaer K.W, 2005 | Family1 VI4 |
|  | S35 | c.192_212dup | p.A65_A71dup | PolyA | Normal | Clinodactyly B4, Camptodactyly B3 | | 4 | Kjaer K.W, 2005 | Family1 VI7 |
|  | S36 | c.192_212dup | p.A65_A71dup | PolyA | Normal | Synpolydactyly B5/6, Camptodactyly R4 | | 3 | Kjaer K.W, 2005 | Family1 VI14 |
|  | S37 | c.192_212dup | p.A65_A71dup | PolyA | Clinodactyly L5 | Synpolydactyly B5/6 | | 5 | Kjaer K.W, 2005 | Family1 VI15 |
|  | S38 | c.192_212dup | p.A65_A71dup | PolyA | Syndactyly B3/4 | Normal | | 2 | Kjaer K.W, 2005 | Family1 VII17 |
|  | S39 | c.192_212dup | p.A65_A71dup | PolyA | Brachydactyly B1,5 | Synpolydactyly R5/6 | | 4 | Kjaer K.W, 2005 | Family1 VIII9 |
|  | S40 | c.192_212dup | p.A65_A71dup | PolyA | Syndactyly B3/4 | Synpolydactyly R5/6 | | 4 | Kjaer K.W, 2005 | Family1 VIII10b |
|  | S41 | c.192_212dup | p.A65_A71dup | PolyA | Syndactyly B3/4 | Normal | | 2 | Kjaer K.W, 2005 | Family1 VII18 |
|  | S42 | c.192_212dup | p.A65_A71dup | PolyA | Brachydactyly R1 | Normal | | 1 | Kjaer K.W, 2005 | Family1 VIII11 |
|  | S43 | c.192_212dup | p.A65_A71dup | PolyA | Syndactyly B3/4 | Normal | | 2 | Kjaer K.W, 2005 | Family1 VII20 |
|  | S44 | c.192_212dup | p.A65_A71dup | PolyA | Clinodactyly R5, Brachydactyly B1,2,5 | Normal | | 3 | Kjaer K.W, 2005 | Family1 VIII12 |
|  | S45 | c.192_212dup | p.A65_A71dup | PolyA | Syndactyly B3/4 | Normal | | 2 | Kjaer K.W, 2005 | Family1 VII23 |
|  | S46 | c.192_212dup | p.A65_A71dup | PolyA | Brachydactyly R5 | Normal | | 1 | Kjaer K.W, 2005 | Family1 VII28 |
|  | S47 | c.192_212dup | p.A65_A71dup | PolyA | Synpolydactyly L3/4 | Normal | | 2 | Kjaer K.W, 2005 | Family1 VIII16b |
| 12 | S48 | c.189_212dup | p.A64_A71dup | PolyA | Syndactyly L3/4, Synpolydactyly R3/4 | Synpolydactyly B5/6 | | 7 | Our study (Family F) | IV4 |
|  | S49 | c.189_212dup | p.A64_A71dup | PolyA | Synpolydactyly B3/4, Clinodactyly B5 | Synpolydactyly B5/6 | | 10 | Our study (Family F) | III6 |
| 13 | S50 | c.189_212dup | p.A64_A71dup | PolyA | Clinodactyly B4/5 | Synpolydactyly B5/6 | | 6 | Xin, Q. 2012 | V1 |
|  | S51 | c.189_212dup | p.A64_A71dup | PolyA | Clinodactyly B5, Brachydactyly B2 | Synpolydactyly B5/6 | | 8 | Xin, Q. 2012 | V2 |
|  | S52 | c.189_212dup | p.A64_A71dup | PolyA | Synpolydactyly B3/4, Camptodactyly B5 | Synpolydactyly B5/6 | | 10 | Xin, Q. 2012 | III2 |
|  | S53 | c.189_212dup | p.A64_A71dup | PolyA | Clinodactyly L4 | Brachydactyly B5 | | 3 | Xin, Q. 2012 | IV2 |
| 14 | S54 | c.189_212dup | p.A64_A71dup | PolyA | Syndactyly L3/4, Clinodactyly R4 | Synpolydactyly L5/6 | | 4 | Dai, L. 2005 | V25 |
|  | S55 | c.189_212dup | p.A64_A71dup | PolyA | Synpolydactyly B3/4 | Normal | | 4 | Dai, L. 2005 | IV20 |
| 15 | S56 | c.189_212dup | p.A64_A71dup | PolyA | Syndactyly R3/4 | Synpolydactyly B5/6 | | 5 | Zaib, T. 2019 | V1 |
|  | S57 | c.189_212dup | p.A64_A71dup | PolyA | Synpolydactyly B3/4, Camptodactyly B5 | Synpolydactyly B5/6 | | 10 | Zaib, T. 2019 | IV2 |
|  | S58 | c.189_212dup | p.A64_A71dup | PolyA | Synpolydactyly B3/4, Camptodactyly B5 | Synpolydactyly B5/6 | | 8 | Zaib, T. 2019 | III1 |
|  | S59 | c.189_212dup | p.A64_A71dup | PolyA | Synpolydactyly B3/4, Camptodactyly B5 | Brachydactyly B4/5 | | 8 | Zaib, T. 2019 | II3 |
|  | S60 | c.189_212dup | p.A64_A71dup | PolyA | Clinodactyly L5, Camptodactyly R5 | Camptodactyly R1 | | 3 | Zaib, T. 2019 | IV11 |
|  | S61 | c.189_212dup | p.A64_A71dup | PolyA | Synpolydactyly L3/4 | Synpolydactyly R5/6 | | 3 | Zaib, T. 2019 | III7 |
| 16 | S62 | c.186_212dup | p.A63_A71dup | PolyA | Synpolydactyly B3/4 | Synpolydactyly B5/6 | | 8 | Our study (Family G) | IV2 |
|  | S63 | c.186_212dup | p.A63_A71dup | PolyA | Synpolydactyly B3/4, Camptodactyly B2/5 | Normal | | 6 | Our study (Family G) | III2 |
| 17 | S64 | c.186_212dup | p.A63_A71dup | PolyA | Syndactyly L3/4, Synpolydactyly R3/4, Camptodactyly B2/5 | Synpolydactyly R5/6 | | 7 | Our study (Family H) | IV1 |
|  | S65 | c.186_212dup | p.A63_A71dup | PolyA | Synpolydactyly B3/4, Clinodactyly B2/5 | Normal | | 6 | Our study (Family H) | III2 |
| 18 | S66 | c.186_212dup | p.A63_A71dup | PolyA | Synpolydactyly B3/4 | Synpolydactyly L5/6, Brachydactyly R4/5 | | 7 | Our study (Family I) | IV1 |
|  | S67 | c.186_212dup | p.A63_A71dup | PolyA | Synpolydactyly B3/4, Clinodactyly B5 | Normal | | 6 | Our study (Family I) | III2 |
| 19 | S68 | c.186_212dup | p.A63_A71dup | PolyA | Syndactyly R3/4 | Synpolydactyly B5/6 | | 5 | Our study (Family J) | IV1 |
|  | S69 | c.186_212dup | p.A63_A71dup | PolyA | Syndactyly R3/4 | Brachydactyly B5 | | 3 | Our study (Family J) | III2 |
|  | S70 | ND | ND | ND | Synpolydactyly R3/4, Camptodactyly R5 | Brachydactyly B5 | | 5 | Our study (Family J) | II4 |
| 20 | S71 | c.186_212dup | p.A63_A71dup | PolyA | Synpolydactyly B3/4, Camptodactyly B2-5 | Synpolydactyly B5/6 | | 10 | Gong, L. 2011 | Patient 3-1 |
|  | S72 | c.186_212dup | p.A63_A71dup | PolyA | Synpolydactyly B3/4, Camptodactyly B2-5 | Synpolydactyly R5/6, Brachydactyly L2-5 | | 9 | Gong, L. 2011 | Patient 2-1 |
|  | S73 | c.186_212dup | p.A63_A71dup | PolyA | Synpolydactyly R3/4, Camptodactyly B2-5 | Synpolydactyly R5/6, Brachydactyly L2-5 | | 7 | Gong, L. 2011 | Patient 1-1 |
| 21 | S74 | c.186_212dup | p.A63_A71dup | PolyA | Synpolydactyly B3/4, Camptodactyly B5 | Synpolydactyly B5/6 | | 10 | Jin, H. 2011 | IV14 |
|  | S75 | c.186_212dup | p.A63_A71dup | PolyA | Synpolydactyly B3/4, Camptodactyly B5 | Synpolydactyly B5/6 | | 10 | Jin, H. 2011 | III15 |
|  | S76 | c.186_212dup | p.A63_A71dup | PolyA | Syndactyly B3/4, Camptodactyly B5 | Synpolydactyly B5/6 | | 8 | Jin, H. 2011 | III11 |
|  | S77 | c.186_212dup | p.A63_A71dup | PolyA | Synpolydactyly B3/4, Camptodactyly B5 | Synpolydactyly B5/6 | | 10 | Jin, H. 2011 | V3 |
| 22 | S78 | c.186_212dup | p.A63_A71dup | PolyA | Clinodactyly B5 | Normal | | 2 | Kjaer, K.W. 2002 | VIII1 |
|  | S79 | c.186_212dup | p.A63_A71dup | PolyA | Syndactyly R3/4/5, Camptodactyly R2,L5 | Synpolydactyly B5/6 | | 6 | Kjaer, K.W. 2002 | VII1 |
|  | S80 | c.186_212dup | p.A63_A71dup | PolyA | Syndactyly L3/4, Clinodactyly R5, Camptodactyly R2/3,L4/5, Brachydactyly R4 | Synpolydactyly B4/5/6 | | 9 | Kjaer, K.W. 2002 | IX-1 |
|  | S81 | c.186_212dup | p.A63_A71dup | PolyA | Syndactyly B3/4, Camptodactyly B3/4/5 | Synpolydactyly R4/5, Camptodactyly L4, Brachydatyly L5 | | 7 | Kjaer, K.W. 2002 | 1X-2 |
|  | S82 | c.186_212dup | p.A63_A71dup | PolyA | Synpolydactyly L5/6, Camptodactyly L2/5 | Normal | | 3 | Kjaer, K.W. 2002 | VIII-5 |
|  | S83 | c.186_212dup | p.A63_A71dup | PolyA | Synpolydactyly B3/4, Camptodactyly B2 | Normal | | 6 | Kjaer, K.W. 2002 | VII-2 |
| 23 | S84 | c.186_212dup | p.A63_A71dup | PolyA | Synpolydactyly B3/4, Clinodactyly B5 | Synpolydactyly B5/6 | | 10 | Zhao, X. 2005 | IV9 |
|  | S85 | c.186_212dup | p.A63_A71dup | PolyA | Synpolydactyly B3/4, Clinodactyly B5 | Synpolydactyly R5/6 | | 8 | Zhao, X. 2005 | III9 |
|  | S86 | c.186_212dup | p.A63_A71dup | PolyA | Synpolydactyly R3/4, Clinodactyly B5 | Normal | | 4 | Zhao, X. 2005 | III14 |
|  | S87 | c.186_212dup | p.A63_A71dup | PolyA | Synpolydactyly B3/4, Clinodactyly B5 | Synpolydactyly B5/6 | | 10 | Zhao, X. 2005 | II3 |
| 24 | S88 | c.186_212dup | p.A63_A71dup | PolyA | Clinodactyly B4, Camptodactyly B5 | Normal | | 4 | Li, Y. 2015 | III9 |
|  | S89 | c.186_212dup | p.A63_A71dup | PolyA | Syndactyly B3/4, Camptodactyly B5 | Polydactyly B5/6 | | 6 | Li, Y. 2015 | II4 |
| 25 | S90 | c.186_212dup | p.A63_A71dup | PolyA | Synpolydactyly R3/4, Clinodactyly B5 | Synpolydactyly B5/6 | | 8 | Akarsu, A.N. 1996 | SPD-3 subject 94 |
|  | S91 | c.186_212dup | p.A63_A71dup | PolyA | Polydactyly B5/6 | Polydactyly L5/6 | | 3 | Akarsu, A.N. 1996 | SPD-4 subject 59 |
| 26 | S92 | c.186_212dup | p.A63_A71dup | PolyA | Syndactyly B3/4 | Syndactyly R4/5, L3/4 | | 4 | Kjaer, K.W. 2005 | Family2 VIII11 |
|  | S93 | c.186_212dup | p.A63_A71dup | PolyA | Synpolydatyly B3/4, Brachydactyly B2,5 | Synpolydactyly B5/6 | | 10 | Kjaer, K.W. 2005 | Family2 VII10 |
|  | S94 | c.186_212dup | p.A63_A71dup | PolyA | Brachydactyly B1,5 | Syndactyly B2/3/4 | | 4 | Kjaer, K.W. 2005 | Family2 VIII15 |
|  | S95 | c.186_212dup | p.A63_A71dup | PolyA | Brachydactyly R1,4, L1 | Normal | | 2 | Kjaer, K.W. 2005 | Family2 VII16 |
|  | S96 | c.186_212dup | p.A63_A71dup | PolyA | Syndactyly B3/4, Brachydactyly B1,5 | Syndatyly L4/5, Synpolydatyly R5/6 | | 7 | Kjaer, K.W. 2005 | Family2 VIII16 |
|  | S97 | c.186_212dup | p.A63_A71dup | PolyA | Synpolydactyly B3/4, Camptodactyly B2-5 | Synpolydactyly B5/6 | | 10 | Kjaer, K.W. 2005 | Family2 VII18b |
|  | S98 | c.186_212dup | p.A63_A71dup | PolyA | Syndactyly L3/4, Camptodactyly B5 | Clinodactyly B4 | | 5 | Kjaer, K.W. 2005 | Family2 VIII27b |
|  | S99 | c.186_212dup | p.A63_A71dup | PolyA | Syndactyly L3/4, Camptodactyly B5 | Clinodactyly B4 | | 5 | Kjaer, K.W. 2005 | Family2 VII28 |
|  | S100 | c.186_212dup | p.A63_A71dup | PolyA | Syndactyly B3/4, Camptodactyly B3/4/5 | Syndactyly R4/5, L2/3 | | 6 | Kjaer, K.W. 2005 | Family2 VII29b |
|  | S101 | c.186_212dup | p.A63_A71dup | PolyA | Brachydactyly B1 | Normal | | 2 | Kjaer, K.W. 2005 | Family2 VIII2a |
|  | S102 | c.186_212dup | p.A63_A71dup | PolyA | Synpolydactyly B3/4 | Normal | | 4 | Kjaer, K.W. 2005 | Family2 IX2a |
|  | S103 | c.186_212dup | p.A63_A71dup | PolyA | Synpolydactyly B3/4, Brachydatyly L2 | Synpolydactyly B5/6 | | 9 | Kjaer, K.W. 2005 | Family2 VIII5a |
|  | S104 | c.186_212dup | p.A63_A71dup | PolyA | Synpolydactyly L3/4, Brachydatyly R3/4/5, L5 | Normal | | 4 | Kjaer, K.W. 2005 | Family2 VIII8a |
|  | S105 | c.186_212dup | p.A63_A71dup | PolyA | Synpolydactyly L3/4, Brachydatyly R3/4/5, L5 | Normal | | 4 | Kjaer, K.W. 2005 | Family2 IX7 |
|  | S106 | c.186_212dup | p.A63_A71dup | PolyA | Synpolydatyly B3/4/5, Brachydactyly L1 | Syndactyly L4/5, Synpolydactyly R5/6 | | 6 | Kjaer, K.W. 2005 | Family2 VIII31 |
| 27 | S107 | c.186_212dup | p.A63_A71dup | PolyA | Syndactyly B3/4, Camptodactyly B2, Brachydactyly B1,5 | Brachydactyly B2/3 | | 8 | Kjaer, K.W. 2005 | Family3 IV11 |
|  | S108 | c.186_212dup | p.A63_A71dup | PolyA | Syndactyly B3/4 | Normal | | 2 | Kjaer, K.W. 2005 | Family3 V7b |
|  | S109 | c.186_212dup | p.A63_A71dup | PolyA | Syndactyly R3/4, L3/4/5, Clinodactyly R5, Brachydactyly B1 | Polydactyly B5/6, Clinodactyly B5 | | 9 | Kjaer, K.W. 2005 | Family3 VI3 |
| 28 | S110 | c.186_212dup | p.A63_A71dup | PolyA | Polydactyly L5/6, Synpolydactyly B3/4, Clinodactyly R5, Brachydactyly B1 | Polydactyly B5/6 | | 10 | Kjaer, K.W. 2005 | Family4 V1 |
| 29 | S111 | c.204G>C(reported as c.291C>T) | p.A68A (reported as p.A60A) | PolyA | Polydactyly R1/2 | Normal | | 1 | Liu, Z. 2015 | Patient A |
|  | S112 | c.204G>C(reported as c.291C>T) | p.A68A (reported as p.A60A) | PolyA | Polydactyly R1/2 | Normal | | 1 | Liu, Z. 2015 | Patient B |
|  | S113 | c.204G>C(reported as c.291C>T) | p.A68A (reported as p.A60A) | PolyA | Polydactyly B1/2 | Normal | | 2 | Liu, Z. 2015 | Patient C |
|  | S114 | c.204G>C(reported as c.291C>T) | p.A68A (reported as p.A60A) | PolyA | Polydactyly B1/2 | Polydactyly B5/6 | | 4 | Liu, Z. 2015 | Patient D |
| 30 | S115 | c.323–336del | p.P108Rfs*124 | Non-homeobox | Normal | Synpolydactyly B1/2, B4/5 | | 4 | Goodman, F. 1998 | Pedigree1 V4 |
|  | S116 | c.323–336del | p.P108Rfs*124 | Non-homeobox | Syndactyly B3/4 | Syndactyly R4/5 | | 3 | Goodman, F. 1998 | Pedigree1 VI1 |
| 31 | S117 | c.556C>T | p.R186* | Non-homeobox | Normal | Polydactyly B5/6 | | 2 | Wang, B. 2017 | IV1 |
|  | S118 | c.556C>T | p.R186* | Non-homeobox | Clinodactyly B5 | Polydactyly R5/6 | | 3 | Wang, B. 2017 | III2 |
|  | S119 | c.556C>T | p.R186* | Non-homeobox | Clinodactyly B5 | Normal | | 2 | Wang, B. 2017 | II2 |
| 32 | S120 | c.610delC | p.G205Afs*61 | Non-homeobox | Clinodactyly B5 | Synpolydactyly B5/6 | | 6 | Our study (Ni, F.2018) | IV1 |
| 33 | S121 | c.683G>T (reported as c.659G>T) | p.G228V (reported as p.G220V) | Non-homeobox | Syndactyly B3/4, Clinodactyly B5, Camptodactyly B5 | Normal | | 6 | Fantini, S. 2009 | III15 |
| 34 | S122 | c.683G>C (reported as c.659G>C) | p.G228A (reported as p.G220A) | Non-homeobox | Syndactyly B3/4, Clinodactyly B5 | Normal | | 4 | Zhou, X. 2013 | III2 |
|  | S123 | c.683G>C (reported as c.659G>C) | p.G228A (reported as p.G220A) | Non-homeobox | Syndactyly B3/4, Clinodactyly B5 | Normal | | 4 | Zhou, X. 2013 | II4 |
|  | S124 | c.683G>C (reported as c.659G>C) | p.G228A (reported as p.G220A) | Non-homeobox | Syndactyly B2/3/4 | Normal | | 2 | Zhou, X. 2013 | III4 |
|  | S125 | c.683G>C (reported as c.659G>C) | p.G228A (reported as p.G220A) | Non-homeobox | Syndactyly B3/4, Clinodactyly B5 | Normal | | 4 | Zhou, X. 2013 | II7 |
| 35 | S126 | c.708delC | p.N236Kfs*30 | Non-homeobox | Synpolydactyly B3/4 | Polydactyly B1/2 | | 6 | Radhakrishnan, P. 2017 | Mother |
| 36 | S127 | c.742C>T | p.Q248* | Non-homeobox | Clinodactyly B5 | Syndactyly R4/5 | | 4 | Kurban, M. 2011 | J |
|  | S128 | c.742C>T | p.Q248* | Non-homeobox | Normal | Brachydactyly B5 | | 2 | Kurban, M. 2011 | K |
|  | S129 | c.742C>T | p.Q248* | Non-homeobox | Clinodactyly B4 | Syndactyly B3/4/5 | | 4 | Kurban, M. 2011 | I |
| 37 | S130 | c.781+1G>A | p.G190fs*4 | Non-homeobox | Synpolydactyly B3/4 | Clinodactyly B1 | | 6 | Shi, X. 2013 | IV2 |
|  | S131 | c.781+1G>A | p.G190fs*4 | Non-homeobox | Syndactyly R3/4 | Synpolydactyly R1/2, B5/6 | | 3 | Shi, X. 2013 | III2 |
|  | S132 | c.781+1G>A | p.G190fs*4 | Non-homeobox | Syndactyly R3/4 | Syndactyly R4/5, Clinodactyly B1 | | 4 | Shi, X. 2013 | III3 |
| 38 | S133 | c.782_2delA (reported as c.758_2delA ) |  | Non-homeobox | Syndactyly B3/4, Clinodactyly B5 | Normal | | 4 | Kan, S.H. 2003 | III1 |
|  | S134 | c.782_2delA (reported as c.758_2delA ) |  | Non-homeobox | Clinodactyly B5 | Normal | | 2 | Kan, S.H. 2003 | II3 |
|  | S135 | c.782_2delA (reported as c.758_2delA ) |  | Non-homeobox | Clinodactyly B5 | Normal | | 2 | Kan, S.H. 2003 | I2 |
| 39 | S136 | c.820C>T | p.R274* | Non-homeobox | Camptodactyly B1-5, Brachydactyly B1, 5 | Normal | | 4 | Jamsheer, A. 2012 | Proband |
|  | S137 | c.820C>T | p.R274* | Non-homeobox | Camptodactyly B5, Brachydactyly B5 | Brachydactyly B5 | | 6 | Jamsheer, A. 2012 | Proband’s father |
| 40 | S138 | c.834delG | p.K279Rfs*42 | Homeobox | Normal | Synpolydactyly B1/2, B4/5, Brachydactyly B2-5 | | 6 | Goodman, F. 1998 | Pedigree2 II4 |
|  | S139 | c.834delG | p.K279Rfs*42 | Homeobox | Normal | Synpolydactyly B1/2, B5/6 | | 8 | Goodman, F. 1998 | Pedigree2 IV4 |
| 41 | S140 | c.859C>T | p.Q287* | Homeobox | Normal | Polydactyly B1/2 | | 2 | Guo, X. 2019 | IV1 |
|  | S141 | c.859C>T | p.Q287* | Homeobox | Clinodactyly B5 | Clinodactyly B2 | | 4 | Guo, X. 2019 | III4 |
| 42 | S142 | c.916C>T (reported as c.892C>T) | p.R306W (reported as p.R298W) | Homeobox | Clinodactyly B5 | Polydactyly L1/2, Brachydactyly B2/3 | | 5 | Debeer P, 2002 | Family 1 IV6 |
| 43 | S143 | c.916C>G | p.R306G | Homeobox | Syndactyly B3/4 | Normal | | 2 | Dai, L. 2014 | Family B I2 |
|  | S144 | c.916C>G | p.R306G | Homeobox | Camptodactyly L3/4,R3/4/5 | Normal | | 2 | Dai, L. 2014 | Family B III1 |
|  | S145 | c.916C>G | p.R306G | Homeobox | Clinodactyly R3/4 | Normal | | 1 | Dai, L. 2014 | Family B I2 |
|  | S146 | c.916C>G | p.R306G | Homeobox | Synpolydactyly L5/6 | Normal | | 2 | Dai, L. 2014 | Family B I2 |
| 44 | S147 | c.917G>A (reported as c.893G>A) | p.R306Q (reported as p.R298Q) | Homeobox | Synpolydactyly B3/4 | Normal | | 4 | Wang, B. 2012 | III G22 |
|  | S148 | c.917G>A (reported as c.893G>A) | p.R306Q (reported as p.R298Q) | Homeobox | Clinodactyly B2 | Normal | | 2 | Wang, B. 2012 | III G21 |
| 45 | S149 | c.917G>A | p.R306Q | Homeobox | Synpolydactyly B3/4 | Synpolydactyly R5/6 | | 6 | Ma, D. 2016 | II2 |
| 46 | S150 | c.917G>A | p.R306Q | Homeobox | Syndactyly B3/4 | Normal | | 2 | Dai, L. 2014 | FA－IV26 |
|  | S151 | c.917G>A | p.R306Q | Homeobox | Normal | Clinodactyly L1/2 | | 1 | Dai, L. 2014 | FA－III3 |
|  | S152 | c.917G>A | p.R306Q | Homeobox | Normal | Syndactyly L2/3 | | 1 | Dai, L. 2014 | FA－III22 |
|  | S153 | c.917G>A | p.R306Q | Homeobox | Syndactyly B3/4 | Normal | | 2 | Dai, L. 2014 | FA－V9 |
| 47 | S154 | c.917G>A | p.R306Q | Homeobox | Syndactyly B3/4 | Normal | | 2 | Deng, H. 2017 | Generation III member2 |
| 48 | S155 | c.925A>T | p.I309F | Homeobox | Normal | Polydactyly L5/6 | | 1 | Our study (Family K) | IV5 |
|  | S156 | c.925A>T | p.I309F | Homeobox | Normal | Camptodactyly R4 | | 1 | Our study (Family K) | III8 |
|  | S157 | c.925A>T | p.I309F | Homeobox | Normal | Polydactyly B5/6 | | 2 | Our study (Family K) | III7 |
|  | S158 | c.925A>T | p.I309F | Homeobox | Normal | Polydactyly L1/2 | |  | Our study (Family K) | III14 |
|  | S159 | c.925A>T | p.I309F | Homeobox | Normal | Polydactyly L5/6 | |  | Our study (Family K) | III16 |
|  | S160 | c.925A>T | p.I309F | Homeobox | Normal | Polydactyly L5/6 | | 1 | Our study (Family K) | II2 |
| 49 | S161 | c.947C>G (reported as c.923C>G) | p.S316C (reported as p.S308C) | Homeobox | Brachydactyly L5 | Normal | | 1 | Johnson, D. 2003 | Family C III2 |
| 50 | S162 | c.964A>C (reported as c.940A>C) | p.I322L (reported as p.I314L) | Homeobox | Brachydactyly B3/4/5 | Brachydactyly B2-5 | | 4 | Caronia, G. 2003 | VI.2 |
|  | S163 | c.964A>C (reported as c.940A>C) | p.I322L (reported as p.I314L) | Homeobox | Brachydactyly B2/4 | Brachydactyly B2-5 | | 4 | Caronia, G. 2003 | V.2 |
|  | S164 | c.964A>C (reported as c.940A>C) | p.I322L (reported as p.I314L) | Homeobox | Synpolydactyly B3/4, Brachydactyly B2-5 | Normal | | 6 | Caronia, G. 2003 | VI.7 |
| 51 | S165 | c.964A>C (reported as c.940A>C) | p.I322L (reported as p.I314L) | Homeobox | Clinodactyly B4, Brachydactyly B3 | Normal | | 4 | Johnson, D. 2003 | Family A III13 |
|  | S166 | c.964A>C (reported as c.940A>C) | p.I322L (reported as p.I314L) | Homeobox | Brachydactyly B3/4/5 | Normal | | 2 | Johnson, D. 2003 | Family B IV3 |
|  | S167 | c.964A>C (reported as c.940A>C) | p.I322L (reported as p.I314L) | Homeobox | Normal | Brachydactyly L4 | | 1 | Johnson, D. 2003 | Family B III5 |
|  | S168 | c.964A>C (reported as c.940A>C) | p.I322L (reported as p.I314L) | Homeobox | Synpolydactyly B3/4, Clinodactyly L4, Brachydactyly B3 | Normal | | 5 | Johnson, D. 2003 | Family B II2 |
| 52 | S169 | c.973C>A (reported as c.949C>A) | p.Q325K (reported as p.Q317K) | Homeobox | Camptodactyly B1-4, Brachydactyly B | Syndactyly B, Clinodactyly L, Brachydactyly B | | 9 | Ibrahim, D. M. 2013 | One case(without family history) |
| 53 | S170 | c.974A>G (reported as c.950A>G) | p.Q325R (reported as p.Q317R) | Homeobox | Syndactyly R4/5, Camptodactyly B2/3/4 | Syndactyly B3/4 | | 5 | Zhao, X. 2007 | Family1 V10 |
|  | S171 | c.974A>G (reported as c.950A>G) | p.Q325R (reported as p.Q317R) | Homeobox | Syndactyly B4/5 | Syndactyly B2/3 | | 4 | Zhao, X. 2007 | Family1 IV10 |
|  | S172 | c.974A>G (reported as c.950A>G) | p.Q325R (reported as p.Q317R) | Homeobox | Syndactyly R4/5, Camptodactyly B2/3/4 | Syndactyly B2/3/4 | | 5 | Zhao, X. 2007 | Family1 IV20 |
|  | S173 | c.974A>G (reported as c.950A>G) | p.Q325R (reported as p.Q317R) | Homeobox | Syndactyly B4/5 | Syndactyly B2/3 | | 4 | Zhao, X. 2007 | Family1 V13 |

*B:bilateral. R:right, L:left. †References were cited in supplemental materials 2. ‡ NM000523.4
