# Supplementary figures and images for: A Novel Missense Variant of HOXD13 Caused Atypical Synpolydactyly by Impairing the Downstream Gene Expression and Literature Review for Genotype–Phenotype Correlations
Source: Front Genet. 2021 Oct 27;12:731278. doi: 10.3389/fgene.2021.731278 (PMC8579070; doi:10.3389/fgene.2021.731278)

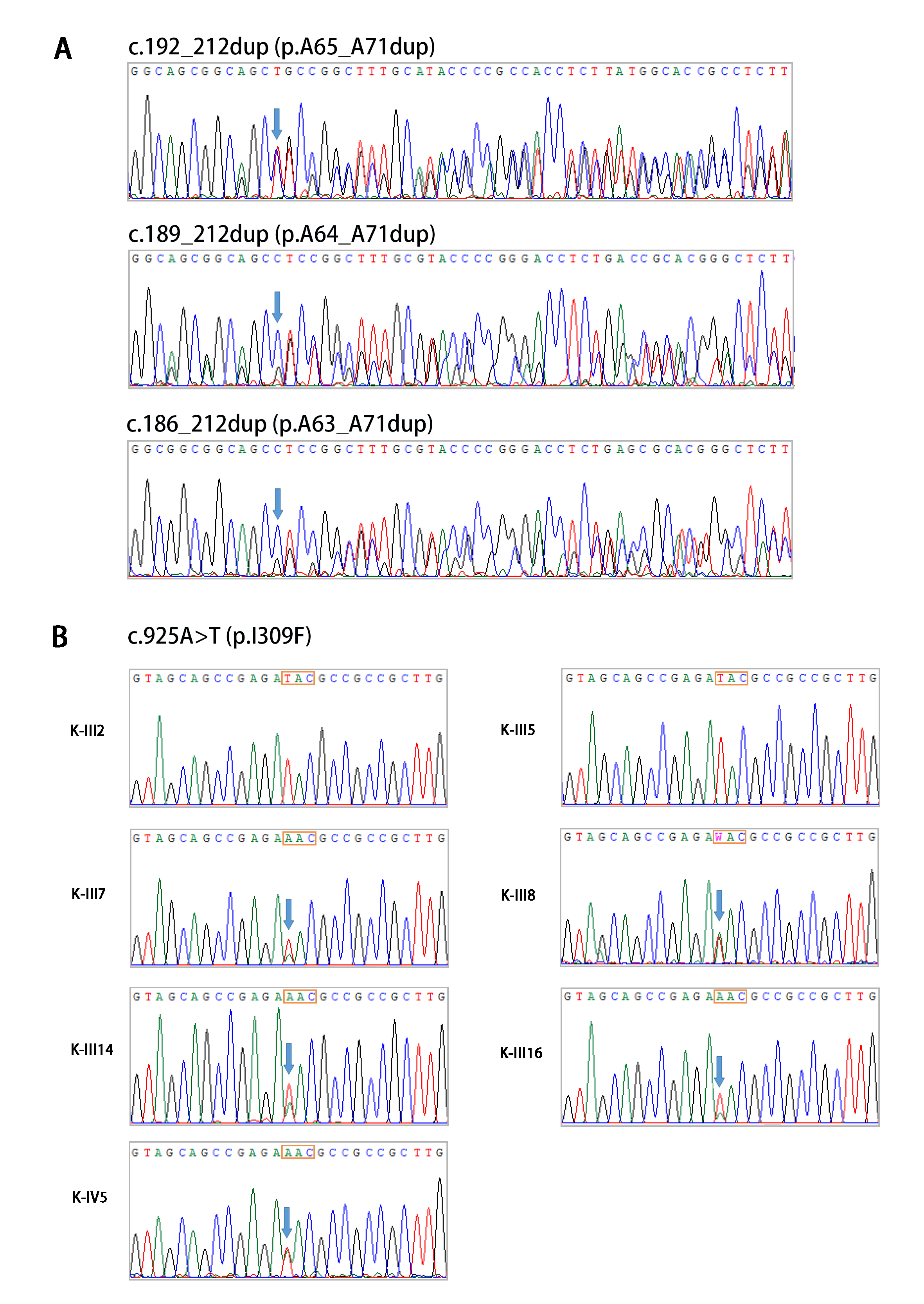

Supplement: Supplementary file 2 [file Image_1.TIF]
